# Case Report: Imaging Features and Successful Management of Ureteral Stenosis in a Kitten With Bilateral Atypical Papillary Transitional Mucosal Hyperplasia

**DOI:** 10.3389/fvets.2021.796638

**Published:** 2022-02-03

**Authors:** Minji Kwon, Sungsoo Kim, Kichang Lee, Hakyoung Yoon

**Affiliations:** ^1^College of Veterinary Medicine, Jeonbuk National University, Jeonbuk, South Korea; ^2^VIP Animal Medical Center, Seoul, South Korea

**Keywords:** feline, ureteral anomaly, congenital ureteral obstruction, hydronephrosis, subcutaneous ureteral bypass

## Abstract

A 3-month-old cat weighing 2.62 kg was brought to the VIP Animal Medical Center with vomiting, diarrhea, anorexia, and depression. Laboratory findings confirmed azotemia with elevated blood urea nitrogen (BUN) (168.9 mg/dl) and creatinine (5.9 mg/dl) and symmetric dimethylarginine (SDMA) (86 μg/dl). Abdominal radiography revealed bilateral renomegaly. Ultrasonography revealed bilateral hydronephrosis and left partial and right complete ureteral obstructions with bilateral focal irregular and circumferential thickenings at ureters. Reduction of bilateral renal function was confirmed through excretory urography on computed tomography. The cat underwent subcutaneous ureteral bypass surgery on the left kidney, right nephrectomy, and bilateral ureterectomy. Histopathology of bilateral ureteral irregular and circumferential thickening revealed bilateral atypical papillary transitional mucosal hyperplasia. Three days after surgery, the left hydronephrosis resolved, and azotemia was almost resolved. This is the first report of successful management of ureteral stenosis with congenital papillary transitional mucosal hyperplasia in a kitten using multimodality imaging.

## Introduction

Feline ureteral obstruction can cause acute kidney injury (AKI) by increasing pressure in the renal pelvis and ureters (1). The most common biochemical abnormality is azotemia, followed by hyperphosphatemia, hypercalcemia, hypocalcemia, and hyperkalemia (2–10). Medical management for AKI and uremic and electrolyte disturbances secondary to ureteral obstruction is essential (11). Relieving the obstructive process as soon as possible is the preferred method for patient stabilization, and there are several surgical methods to treat ureteral obstruction, such as subcutaneous ureteral bypass device (SUB), ureterotomy, ureteral stenting, ureteral reimplantation, ureteronephrectomy, and renal transplantation (2, 11–14). The risk of reocclusion with stent or ureter surgery is ~22%, and preliminary abstract data indicate that ~25% of cats show occlusion of the SUB device, but only 13% require intervention (4, 10, 14). Therefore, regardless of the surgical or interventional option chosen, it is important to indicate that there is a risk of recurrence of the obstruction and that further procedures may be required in the future (4, 10, 14).

Feline ureteral obstruction may be due to congenital causes, such as congenital ureteral stenosis, ureteral ectopia, or acquired causes such as secondary ureteral stenosis, ureterolithiasis, dried solidified blood calculi, iatrogenic ureteral ligation, retroperitoneal fibrosis, and neoplasia (3, 4, 15–20). Ureteral stenosis is the second most common cause of ureteral obstruction after ureterolithiasis in cats (3, 19, 21). It has been reported that most feline ureteral stenosis is caused by the passage of ureteroliths and ureteral surgery (3). Minor predisposing conditions for ureteral stenosis are endogenous or exogenous neoplasms, retroperitoneal fibrosis, circumcaval ureters, and congenital disorders (3).

Feline congenital ureteral obstructions have been reported relatively rarely and may result from a circumscribed narrowing of the ureteral lumen (3). In the veterinary medical literature, the feline congenital ureteral obstruction is reported in five studies (22–26), of which three cases underwent euthanasia, and no case dealt with multimodality imaging description in detail.

This study presents the successful management and the characteristics of histopathologically diagnosed ureteral lesions in a cat with congenital ureteral obstruction and hydronephrosis based on radiography, ultrasonography (US), and computed tomography (CT).

## Case Presentation

A 3-month-old, intact male Korean Short Hair cat weighing 2.62 kg was presented to the VIP Animal Medical Center with vomiting, diarrhea, anorexia, and depression for 4 days. The cat had been diagnosed with parasitic infection 2 days before admission from another hospital and had been on anthelmintic drugs. On physical examination, mild dehydration (5–6%) was observed. Laboratory findings confirmed azotemia with elevated blood urea nitrogen (BUN) (168.9 mg/dl; reference range, 16–33 mg/dl), creatinine (5.9 mg/dl; reference range, 0.6–1.6 mg/dl), and symmetric dimethylarginine (SDMA) (86 μg/dl; reference range, 0–14 μg/dl). Hyperammonemia (221 mg/dl; reference range, 0–95 mg/dl) and elevated feline serum amyloid A (fSAA) (42.4 μg/ml; reference range, 0–14 μg/ml) were also identified. Venous blood gas analysis revealed severe acidosis with pH (7.04; reference range, 7.21–7.41) and electrolyte imbalance with hypernatremia (158 mEq/L; reference range, 149–157), hyperkalemia (5.95 mEq/L, reference range, 3.3–4.5 mEq/L), and hypercalcemia (1.50 mEq/L; reference range, 1.11–1.38 mEq/L). Urinalysis showed low urine specific gravity (USG, 1.005; reference range, >1.035). The possibility of prerenal azotemia due to mild dehydration was considered, and imaging tests were performed to further confirm renal azotemia, such as nephritis and postrenal azotemia due to ureteral obstruction.

**Figure 1:**
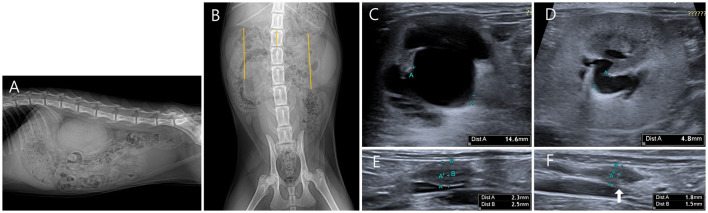
Radiographic and ultrasonography (US) images of the bilateral hydronephrosis in a cat with bilateral ureteral anomalies. **(A)** It is observed that both kidneys overlap below the second to fourth lumbar vertebrae in the right lateral view. **(B)** When the length of the second lumbar vertebra was compared with the bilateral height measured by line in ventrodorsal view, the right kidney (5.51 cm) was measured to be 3.3 times the L2 length (1.53 cm), and the left kidney (5.05 cm) was measured to be 3.6 times the L2 length (1.53 cm). **(C)** A transverse US image shows pyelectasis of the right kidney. The height of the right renal pelvis was measured to be 14.6 mm. **(D)** A transverse US image shows the pyelectasis of the left kidney. The height of the left renal pelvis was measured to be 4.8 mm. **(E)** Focal polypoid mural thickening was observed in the middle of the right ureter. Focally thickened ureteral wall can be seen in circumferential form, and the thickness of the ureteral wall was measured to be 2.3 mm for the dorsal part and 2.5 mm for the ventral part. **(F)** Focal polypoid mural thickening was observed in the middle of the left ureter. Focally thickened ureteral wall can be seen in circumferential form, and the thickness of the ureteral wall was measured to be 1.5 mm for the dorsal part and 1.8 mm for the ventral part.

On the abdominal radiography (Vetter-DR9, MEDIEN, Gyeonggi, Korea), the size of both kidneys was measured to be >3.2 times the length of the second lumbar vertebra, confirming bilateral renomegaly (suggested normal range in healthy cats, 2.1–3.2 times the length of L2) (Figures 1A,B).

On the US (Aplio 300, Canon Medical System Europe B.V., Zoetermeer, Netherlands), focal irregular and circumferential thickening of the ureteral wall was observed in the middle of the bilateral ureters. Thus, ureter dilation and renal pelvic dilation anterior to the site were confirmed. The pyelectasis was more severe on the right side, with 14.6 mm on the right and 4.8 mm on the left in the transverse plane (suggested normal range, ≤3 mm) (Figures 1C–F).

**Figure 2:**
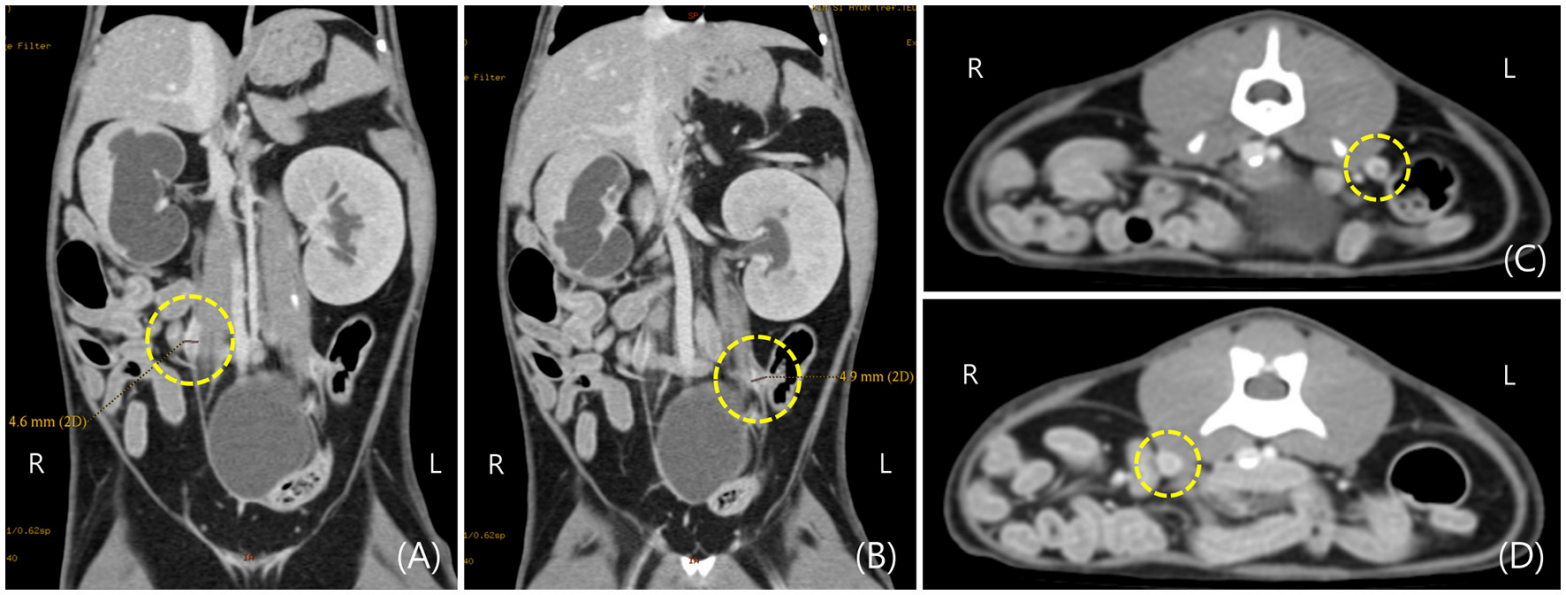
Post-contrast computed tomography (CT) images of hydronephrosis in a cat with bilateral ureteral anomalies. **(A,B)** Focal thickening of the right and left ureteral wall was observed on the dorsal plane of CT image in the area indicated by the dotted circle. **(C,D)** Focal thickening of the right and left ureteral wall was observed on the transverse plane of the CT image in the area indicated by the dotted circle.

Since spontaneous urination was confirmed until daytime at admission to the hospital, it was not an oliguria state. Therefore, CT (Brivo CT385, GE Healthcare, Buckinghamshire, UK) scan and contrast examination were performed to further evaluate the cause of ureter obstruction and renal dysfunction. The CT scan was performed using iohexol (Omnihexol 300, Korea United Pharm Inc., Seoul, South Korea) (800 mg iodine/kg) under general anesthesia with isoflurane. The CT scans were performed before contrast injection and after contrast for 30 s, 3 min, and 10 min. In pre-contrast images, the Hounsfield unit (HU) of the focally thickened ureters was 30–45. After contrast injection, peripheral rim enhancement (HU: 140–180) was observed on the lesion after intravenous contrast injection (Figure 2).

Post-contrast images confirmed that filtration from the bilateral renal cortex to the medulla was not normally performed for 10 min even after injection of contrast medium, and bilateral renal pelvic dilation was also observed on CT images.

Therefore, considering the comprehensive imaging findings, the possibility of secondary hydroureteronephrosis due to ureteral stenosis caused by bilateral polypoid nodules was highly evaluated. Differential diagnosis (DDx) for bilateral ureteral irregular and circumferential thickening and contrast enhancement in the forms of the peripheral rims, ureteral polypoid or papillary transitional epithelial hyperplasia, ureteral granuloma, and ureteral tumor were considered.

Since bilateral renal functions were reduced due to ureteral obstructions, surgical resolution of the obstructions was considered the most effective solution. It recommended bilateral ureterectomy and bilateral SUB surgery; however, the owner wanted single-sided SUB due to financial constraints. It was decided to remove the right kidney, which was observed as severe hydronephrosis compared to the left. Anesthesia was induced with butorphanol (Butophan Inj., Myung Moon Pharma, Seoul, South Korea) (0.2 mg/kg IV) and midazolam (Midazolam Inj., Bukwang Pharma, Seoul, South Korea) (0.1 mg/kg IV). Then, anesthesia was induced with propofol (Anepol, Hana Pharma, Hwasung, South Korea) (6 mg/kg IV), intubated, and maintained with isoflurane (Foran solution, Choongwae Pharma, Seoul, South Korea) in 100% oxygen. The focally thickened lesion in the left ureter was removed locally, and the left renal pelvis and urinary bladder were surgically connected through SUB. The right side was excised from the kidney to the ureteral lesion.

**Figure 3:**
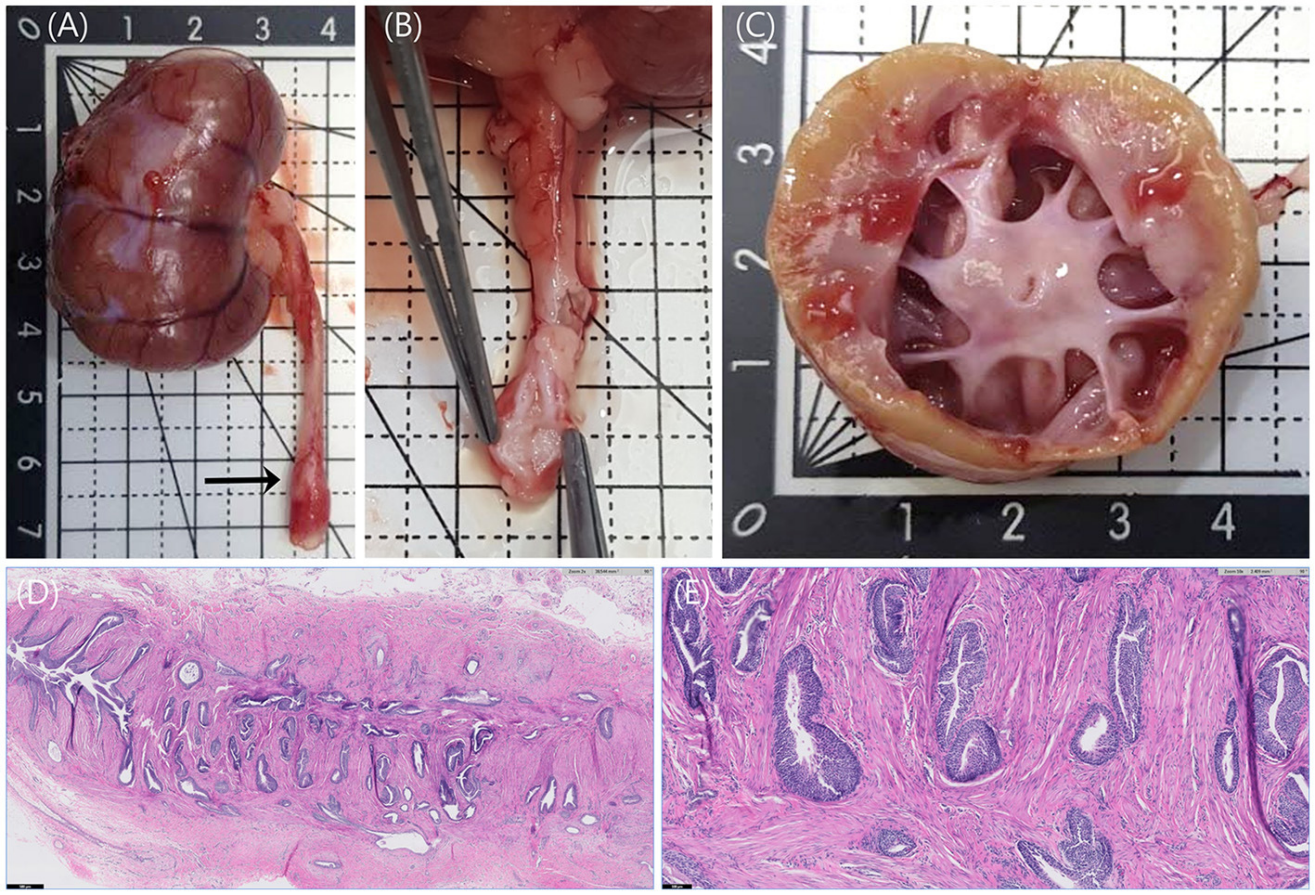
Anatomy of right kidney and right ureter and histopathology of right ureter stained with hematoxylin and eosin (H&E) staining. **(A)** The enlarged right kidney and focally thickened ureter (arrow) were observed. **(B)** The surface of the thickened ureteral wall was irregularly protruded. **(C)** When the right kidney was dissected, the renal pelvis was severely dilated, and the cortex was yellowish, unlike the normal reddish-brown color. **(D)** Numerous invaginated folds of uroepithelium are observed at the right ureteral malformation. H&E, ×20, Scale bar = 500 μm. **(E)** Invaginated folds and embedded nests of uroepithelium appear to be entrapped within the smooth muscle layer of the ureter. No epithelial dysplasia is identified. H&E, × 100, Scale bar = 100 μm.

On gross examination, the right kidney was enlarged. When the kidney was dissected, the renal pelvis was severely dilated, and it was found that the cortex degenerated to yellowish (Figures 3A,C). In addition, focal nodular thickenings were observed in the middle parts of the bilateral ureters. When the areas were cut in half, they found that the surfaces of the lumens were irregularly protruded (Figures 3A,B).

The right kidney and bilateral ureteral lesions were histologically analyzed in the laboratory. Moderate hydronephrosis with mild lymphoplasmacytic interstitial nephritis, mild renal nephron atrophy, and interstitial fibrosis, and few retained fetal glomeruli were confirmed in the right kidney. Microscopic findings from the hematoxylin and eosin (H&E) staining were consistent with bilateral atypical papillary hyperplasia and folding of the transitional epithelium of the ureter (Figures 3D,E). This transitional epithelium is non-neoplastic and forms invaginated folds that appear to extend into the muscularis layer of the ureteral wall. No epithelial dysplasia was observed. The cross-sections of the ureter were histologically similar with the right side being more severely affected than the left. This is highly likely to be the primary congenital abnormality resulting in secondary obstruction and hydroureteronephrosis.

**Figure 4:**
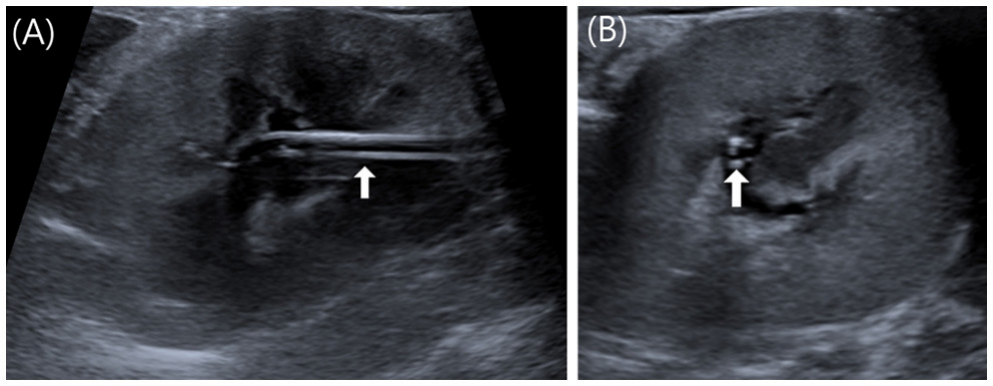
Postoperative US images of left kidney. **(A)** Sagittal image. **(B)** Transverse image. Three days after surgery, the left renal pelvic height was observed to be within 2 mm on US, and a nephrostomy tube was observed normally as two parallel lines in the renal pelvis (white arrow).

Metronidazole (Metrynal Inj., Dai Han Pharma, Seoul, South Korea) (7.5 mg/kg IV q12 h) and cefazolin (Cefazol Inj., Korus Pharma, Gangwon, South Korea) (25 mg/kg IV q12 h) were administered for the prophylactic management of postoperative infections. Two days after surgery, azotemia was resolved with BUN (20.4 mg/dl; reference range, 16–33 mg/dl), creatinine (1.17 mg/dl; reference range, 0.6–1.6 mg/dl), and SDMA (17 μg/dl; reference range, 0–14 μg/dl). Three days after surgery, the left renal pelvic height was observed to be within 2 mm on US, and a nephrostomy tube was observed normally in the renal pelvis (Figure 4). The cat remained clinically healthy until 16 months after surgery.

## Discussion

Feline congenital ureteral obstruction without evidence of intraluminal obstruction has been rarely documented. To our knowledge, unilateral or bilateral congenital ureteral obstruction has been reported previously in five cats (22–26). Previous reports of considered feline congenital ureteral obstruction included one cat with bilateral ureteral fibrosis without evidence of an underlying cause (22); one cat wherein unilateral double ureteral stenosis without any clinical signs was incidentally discovered on US, although this was not confirmed by histopathology (23); one cat wherein necropsy with bilateral proximal ureteropelvic junction stenosis without fibrosis was confirmed (24); one cat wherein bilateral ureteral strictures with polypoid transitional epithelial hyperplasia and chronic lymphoplasmacytic inflammation were histopathologically revealed (25); and one cat wherein unilateral ureteral stenosis with a normal ureteral epithelium was histopathologically confirmed (26). The age of the five cats that were previously reported to be diagnosed with assumed congenital ureteral obstruction ranged from 4 months to 4 years, and this reported the youngest cat with congenital ureteral obstruction (22–26). This report is the only case of successful management of congenital ureteral stenosis through SUB with multimodality imaging such as radiographic, US, and CT examinations.

Histologically, this case was bilateral papillary ureteral transitional epithelial hyperplasia in the uroepithelium, similar to one previous study (25). However, this case was not accompanied by chronic lymphoplasmacytic inflammation in the uroepithelium. In the five cats with congenital ureteral obstruction described above, only one case of unilateral ureteral stenosis with a normal ureteral epithelium was successfully managed through ureteronephrectomy (26), three cats were euthanized due to end-stage kidney disease associated with bilateral hydronephrosis (22, 24, 25), and one cat was not treated because there were no specific clinical symptoms (23).

Although there are various causes of feline ureteral obstruction, regardless of the etiology, the pathophysiology of obstructive nephropathy on the kidneys is similar (11). When ureteral obstruction occurs, the renal blood flow and glomerular filtration rate (GFR) are reduced, which leads to renal tubular inflammation and injury, resulting in AKI (1). The degree of damage depends on the severity and duration of the obstruction and the presence of pre-existing renal disease (1, 11, 27). The earliest change seen in obstructive nephropathy is the increase in the infiltration of inflammatory cells, with macrophage and T lymphocytes predominantly increasing within 4 h of post-obstruction (28). This pathophysiology may cause renomegaly due to hydronephrosis and/or inflammatory changes, as in this case, and if the obstruction is prolonged, renal atrophy through fibrosis may occur (1, 11, 27, 29, 30). The nephropathy leads to a decrease in renal function, and therefore, the nephrographic phase time of this patient was considered to be delayed in CT scan (31).

Hyperammonemia without recognizable concurrent hepatic disease may result from increased production or excessive ammonia absorption into the venous circulation (32). In humans with normal hepatic function, the most common causes of hyperammonemia are urinary retention and infection with urease-producing bacteria (32–34). Hyperammonemia in this cat may have been caused by urinary stasis due to ureteral obstruction and infection with urease-producing organisms.

The definitive cause of ureteral stenosis is ideally based on histopathological findings related to stenosis of the ureter lumen (3). Since biopsy is not commonly performed in the feline ureter for invasive reasons, imaging tests may be helpful in the diagnosis (3, 25). Abdominal radiographs can help identify renomegaly and radiopaque calculi in the urinary tract, which are important in identifying diseases related to ureteral obstruction (11). However, there are limitations to evaluating the renal parenchyma and structures such as the renal pelvis and ureter using only radiographic examination. Antegrade pyelography provides excellent opacification of the collecting system by injecting a contrast medium into the renal pelvis (35). However, this invasive procedure has risks, such as hemorrhage, focal damage of renal tissue, and secondary infection (36). The abdominal US can help identify hydronephrosis and hydroureter and is useful in identifying the location, pattern, and cause of urinary tract obstruction (2, 25, 26). However, it may be difficult to identify distal ureteral obstruction by US (26), and not all calculi may be confirmed by radiographic and ultrasound examinations (2). Thus, it may be helpful to determine the overall structure through advanced imaging examinations such as CT (2). However, since CT also carries burdens on anesthesia, cost, and radiation exposure, examinations through radiography, US, and CT can be complementary imaging examinations.

A limitation of this case report was that urine culture was not performed prior to antibiotic use. Although no specific organism was detected in the urine culture test the day after the first visit, the effect of the antibiotic used on the first visit or the difficulty of the accurate test due to the low urine specific gravity cannot be excluded (37). Hyposthenuria (urine-specific gravity in young cats <1.017) can be caused by central or nephrogenic diabetes insipidus or conditions that interfere with the concentration of urine, such as hypercalcemia, sepsis, and hepatic disorders (38, 39). The hyposthenuria in this cat may have resulted from interference with the action of antidiuretic hormone (ADH) due to hypercalcemia.

To the best of our knowledge, no cases have reported successful management of bilateral ureteral stenosis by ureteral atypical papillary transitional mucosal hyperplasia with a detailed description of multimodality imaging characteristics. In cases of ureteral papillary nodule obstruction in young cats, atypical papillary transitional mucosal hyperplasia should be included in the differential diagnosis.

## Data Availability Statement

The original contributions presented in the study are included in the article/supplementary material, further inquiries can be directed to the corresponding author/s.

## Ethics Statement

Ethical review and approval was not required for the animal study because authors declare no IACUC or other approval was needed. Written informed consent was obtained from the owners for the participation of their animals in this study.

## Author Contributions

MK and HY: conception, design, and drafting the article. MK, SK, and HY: acquisition of data. MK, KL, and HY: analysis and interpretation of data. All authors contributed to the article, revising article for intellectual content, final approval of the completed article, and approved the submitted version.

## Conflict of Interest

The authors declare that the research was conducted in the absence of any commercial or financial relationships that could be construed as a potential conflict of interest.

## Publisher's Note

All claims expressed in this article are solely those of the authors and do not necessarily represent those of their affiliated organizations, or those of the publisher, the editors and the reviewers. Any product that may be evaluated in this article, or claim that may be made by its manufacturer, is not guaranteed or endorsed by the publisher.

## Abbreviations

BUN: blood urea nitrogen
SDMA: symmetric dimethylarginine
fSAA: feline serum amyloid A
USG: urine specific gravity
US: ultrasonography
EU: excretory urography
CT: computed tomography
SUB: subcutaneous ureteral bypass
DDx: differential diagnosis
H&E: hematoxylin and eosin staining
GFR: glomerular filtration rate
AKI: acute kidney injury
ADH: antidiuretic hormone.
